# Highlights from the International Summit on Intellectual Disability and Dementia Implications for Brazil

**DOI:** 10.1590/1980-57642018dn12-040001

**Published:** 2018

**Authors:** Flavia H. Santos, Karen Watchman, Matthew P. Janicki, International Summit on Intellectual Disability and Dementia

**Affiliations:** 1 São Paulo State University, Bauru, SP, Brazil; 2 University College Dublin, Ireland; 3 University of Stirling, Scotland, UK; 4 University of Illinois at Chicago, USA.

**Keywords:** dementia, Down syndrome, intellectual disability, national plan, summit, demência, síndrome de Down, deficiência intelectual, plano nacional, cúpula

## Abstract

In October of 2016, an interdisciplinary group representing North and South American and European countries met in Glasgow, Scotland, to scrutinize universal issues regarding adults with intellectual disability (ID) affected by dementia and to produce recommendations and guidelines for public policy, practice, and further research. The aim of this paper is to apprise relevant outcomes of the Summit targeting Brazilian researchers, clinicians, and nongovernmental organizations in the field of ageing and dementia that are committed to developing the Brazilian national dementia plan. Three core themes were covered by the Summit: i) human rights and personal resources, ii) personalized services and caregiver support, and iii) advocacy and public impact. The exploration of the themes highlighted variations across countries, and revealed consensual views on matters such as international networks, guidance for practices, and advocacy on behalf of both people with ID affected by dementia, and their families. The authors outline the challenges Brazil must confront regarding ageing and dementia and proffer recommendations to address the needs of adults with ID affected by dementia within this scenario; both of which would help in developing the Brazilian national dementia plan.

## RESUMO

Em outubro de 2016, um grupo interdisciplinar representando países da América do Norte e do Sul, bem como Europeus reuniu-se em Glasgow, na Escócia, para examinar questões relativas aos adultos com deficiência intelectual (DI) afetados por demência e para produzir recomendações e diretrizes referentes às políticas públicas, práticas e pesquisa. O objetivo deste artigo é informar os resultados relevantes da Cúpula aos pesquisadores, clínicos e organizações não-governamentais brasileiras que atuam no campo do envelhecimento e da demência, e estão envolvidos no desenvolvimento do plano nacional de demência. Três temas centrais foram discutidos na Cúpula: i) Direitos humanos e recursos pessoais, ii) Serviços personalizados e apoio aos cuidadores, e iii) Advocacia e impacto público. A exploração dos temas destacou as variações entre os países e revelou visões consensuais em questões como redes internacionais, orientação para práticas e defesa de direitos em nome de pessoas com DI afetadas por demência e suas famílias. Os autores descrevem os desafios que o Brasil deve enfrentar em relação ao envelhecimento e à demência e apresentam recomendações para atender às necessidades de adultos com DI afetados por demência nesse cenário; ambos aspectos contribuem para o desenvolvimento do plano nacional de demência.

In 2004, the APAE São Paulo, in collaboration with UNESCO and the Ministry of Health, organized the first Brazilian Congress on “Ageing and Intellectual Disability: A Silent Emergency”.^1^ This was the first Latin American forum of discussion focused on the elevated risk and earlier onset for dementia among adults with intellectual disability (ID), particularly those with Down syndrome [code 6D85.9; ICD-11].^2^ In fact, the spectrum of ID is extremely heterogeneous, including aetiology-related neuropsychiatric and somatic characteristics, accompanied by brain dysfunctions which escalate many risk factors for dementia.^3^ Since then, has this matter received enough attention? In general, whilst broader issues have been discussed, many of the nuances have yet to be fully explored and later age neuropathologies are only now raising concerns.

Ageing of populations is a growing phenomenon across the globe; in developing countries the ageing process tends to be more progressive and chronologically earlier than that observed in developed countries.^4^ According to the most recent Brazilian census, there are 190,755,799 inhabitants in the country; of this population, some 7.4% are aged 65 or older and of the total, 1.4% have intellectual disability.^5^ Whilst the number of older adults with ID in Brazil is uncertain, it is clearly increasing along with life expectancy; as is, inevitably, the incidence of dementia.^6^^,^ 7 According to The National Health Survey conducted in 2013-2014 to study the prevalence of disability in Brazil, which included persons aged 60 and over within other age bands, the prevalence of ID in the population was 0.8% in 64,348 interviews (95% CI, 0.7; 0.8). The rate was higher among men, with no difference for age group, race/ethnicity, or macro-regions.^8^

Officially, Brazil has policies and legal mechanisms focused on ageing, for instance the Statute for the Elderly (Estatuto do Idoso; Federal Law 10.741/2003; updated by Law 13.466/2017). However, the implementation of the services and facilities are still underdeveloped. The territorial configuration of the Unified Health System (SUS, Sistema Unificado de Saúde) expresses and reproduces regional inequalities in Brazil, mainly between the North-northeast and South-southeast. Regional health policies in the 2000-2016 period resulted in advances such as investment, planning and extension of the service network to reduce inequalities in health.^9^ Consequently, evidence from the International Monetary Fund highlights that inequality declined between and within Brazil’s 27 states in this period.^10^

According to the 2000 Census,^5^ there is a very low average for the years of education among the aged (3.5 years for men and 3.1 years for women). Around 60% of Brazilians aged 75 years and over were illiterate or functionally illiterate. Overall, their income was below the minimum wage (»US$228.00 per month) and still unequal countrywide. For instance, income levels in rural areas represent about 40% of urban incomes. In the North and Northeast regions, however, the income of elderly in rural areas is less than half that for urban regions. These inequalities contribute to risks identified in the earlier genesis of Alzheimer’s disease given that socioeconomic disadvantage in early life is associated with increased prevalence of dementia.^11^

The review by Neumann and Albert^12^ highlighted the lack of investment in research on ageing, workforce development, and coordinated services and support. Only in the last three years have studies regarding dementia costs in Brazil been carried out. Ferretti et al.^13^ estimated direct social costs of health care supported by government funding within the SUS. This cross-sectional and non-randomized estimate concluded that global costs of dementia were US$1,405 per month [~BRL 5,500] per person, with 56.6% attributable to informal care costs; 15.1% to direct medical costs; and the remaining 37.3% to social costs. At the same time, indirect cost progressively increased when stratified into mild, moderate and severe dementia, and exceeds 69% of family income.^14^ Proportionally, these numbers represent a concern of 1.6 million people diagnosed with dementia, but possibly another 1.2 million remain undiagnosed.^15^ These markers, taken together, convey the need for a national dementia plan, i.e. a set of measures aiming to: i) expand awareness on dementia and reduce stigma, ii) create specialized diagnostic centers and reduce the incidence of misdiagnosis, iii) provide integrated care towards excellence in quality of care and better quality of life, and iv) develop educational programs for family and professional carers.^15^

Older adults with ID are immersed in this precarious scenario, with no specialty dementia-related services targeting adults with ID. Such services would be advantageous because this group has peculiarities and needs that differ from prototypic dementia. Matos et al.^16^ carried out a self-report cross-sectional study in a cohort of Brazilian adults with unspecified ID, mainly men aged 36-65 years. Results revealed, on one hand, greater independence on self-care, sphincter control (continence), mobility and locomotion, and on the other, need of supervision and help in communication and social cognition. In a multicenter study by proxy,^17^ family members were interviewed regarding the functioning of adults with ID (the majority were men, mean age 45 years) in comparison with healthy age-matched controls. Carers observed more changes in physical, neurological, and psychiatric areas in the last five years compared with control subjects of the same age.^17^ These studies provide insights on the main ageing characteristics; however, advances in screening for signs and symptoms of dementia in people with ID and nationwide surveys are still necessary for clinical and research purposes.

Overall, adults with ID affected by dementia have intellectual limitations that: i) emerged during childhood, and ii) significantly restrict the person’s ability to successfully participate in normal day-to-day activities such as self-care, communication, going to school or working, and generally functioning with their community. These limitations produce long-term adaptive or functional support needs, and/or eligibility for public support programs that precede the possible, probable, or definitive diagnosis of dementia according to medical standards.^18^ Nevertheless, dementia manifestations may vary within syndromes or aetiologies of ID.^19^ For instance, onset among adults with Down syndrome is typically after the fourth decade and often accompanied by epilepsy.^20^ Among persons with other intellectual disability, the age of onset may be more normative of the general population and similarly preceded by a transitory stage of mild neurocognitive disorder. In both instances, the stages of progression may overlap, have short durations or be accompanied by comorbidities.^19^ As with other adults, memory loss is an early symptom,^21^ although may not be the first indicator as carers will typically note changes in activities of daily living or in personality beforehand.^17^

However, since most adults with ID are low-educated it is difficult to diagnose dementia based on general neuropsychological batteries or conventional dementia screening tools. Instead, the use of dementia-related ID specific scales^22^ is preferable, mainly in combination with the ICD-11^2^ or DSM-5^23^ criteria. The premorbid level of cognitive abilities cannot always be used as a marker of progression due to the vagaries of individual intellectual and conceptual development. In the case of Brazil, it is important to remember that adults with ID still have low autonomy, i.e. reduced social participation, integration in the community, and opportunities for choice-making.^24^ Their working experiences are scarce, sheltered, and sometimes deferred* by pensions,^25^ suggesting a disadvantaged population. Therefore, typical markers of functioning that are usually helpful in the general population to ascertain neurocognitive decline may be unavailable in people with ID. There may also be a lack of specialists within the diagnostic services who understand the confluence of lifelong ID and ageing-related neuropathologies.

## THE INTERNATIONAL SUMMIT ON INTELLECTUAL DISABILITY AND DEMENTIA

As an attempt to increase awareness, visibility and actions on these matters worldwide, the International Summit on Intellectual Disability and Dementia was convened in Glasgow Scotland, on October 13-14, 2016. The Summit aimed to examine and report the current scientific knowledge regarding dementia and ID-related topics that are often unrecognized or understudied. These included such nuanced topics as advanced dementia and end-of-life care, structures for post-diagnostic supports, family caregiver needs and supports, proactive planning, and subjective perspectives on care and impact of dementia.^26^ These universal topics resonate across countries and care systems as they address a range of issues relevant to public health planning, dementia identification and care, and health care schemes and the Summit’s recommendations have general application to local situations (see Table). The present article summarizes the Summit outcomes as an attempt to draw attention of Brazilian researchers, clinicians and nongovernmental organizations in the field of ageing and dementia to address this critical issue.

**Table:** Key recommendations associated with summit topics.

**Human rights and personal resources**	
Human rights and the convention for rights of persons with disabilities	(a) Recognize dementia as an impairment under the CRPD
	(b) Support a human rights approach to self-determination when a person with ID is affected by dementia
	(c) Support increased dialogue and cooperation between ID services and the dementia advocacy and care sectors
Perspectives of persons with intellectual disability	(a) Undertake more research on care determination situations
	(b) Enable greater involvement of self-advocacy groups in dialogues with providers
	(c) Increase efforts on breaking down bias by research and ethics review boards on using persons with ID as informants
**Individualized services and clinical supports**	
Post-diagnostic support	(a) Study the effectiveness of different non-pharmacological interventions
	(b) Develop guidelines for PDS applications by caregivers and support staff
	(c) Research the prevalence and nature of BPSD in adults with ID who develop dementia
Community dementia capable supports	(a) Develop standards of care for community-based services that provide housing and other supports for persons with ID and dementia
	(b) Promote dementia capable living environments in all places called 'home'
	(c) Prevent arbitrary changes in residence via fiat by government authorities
Advanced dementia	(a) Support continued assessment for changes in disease progression
	(b) Encourage research directed at identifying more sensitive clinical tools for recognizing progression to late stage or advanced dementia
	(c) Develop training in, and practice guidelines for, care practices with advanced dementia
End-of-life care	(a) Create a universal practice guideline on end-of-life supports
	(b) Encourage the use of such supports for end-of-life care in home settings
	(c) Recognize variations in what 'home' may be like with respect to end-of-life care
**Advocacy, public impact, and family caregiver issues**	
Terminology and the use of language	(a) Adopt a standardized list of terms for general use by providers and researchers
	(b) Standardize reporting to include key demographic and subject factors
	(c) Promote positive imagery via non-stigmatizing language
Inclusion in national dementia plans and strategies	(a) Include adults with ID in processes that create national plans
	(b) Advocate that governments provide supportive data related to ID for plan development
	(c) Involve 'self-advocates' [persons with ID] in the development or review of policy documents and plans
Family caregivers	(a) Increase family support and enhance access to information and assistance with respect to family's values, beliefs, ethnicity, and circumstances
	(b) Aid in ageing planning so that families are aware of steps to make appropriate decisions according to their own values and, if possible, valuing person with ID's opinion
	(c) Reduce stress and provide resources to avoid and minimize the negative aspects of caregiving

The audience of the Summit’s reports are practitioners, services planners, family members, advocates, and governmental and non-governmental (or third sector) organizations, and hopefully future researchers in this field. Three core themes were scrutinized: (1) *Human rights and personal resources*, (2) *Individualized services and clinical supports*, and (3) *Advocacy, public impact, and family caregiver issues*. These major topics were amplified and are outlined as follows:

## HUMAN RIGHTS AND PERSONAL RESOURCES

### Human rights and the convention for rights of persons with disabilities

The United Nations’ Convention for the Rights of Persons with Disabilities (CRPD),^27^ should be the foundation to promote interaction and learning across the specialty services that attend both persons with dementia and ID. This cohesive approach may ameliorate the attention to dementia-related issues facing people with ID. Three recommendations were made: (a) recognize dementia as an impairment under the CRPD, (b) support a human rights approach to self-determination when a person with ID is affected by dementia, and (c) support increased dialogue and cooperation between ID services and the dementia advocacy and care sectors.

### Perspectives of persons with intellectual disability

People with dementia usually express their own perspectives. By contrast, little is known about perspectives of adults with ID as they might have less opportunities and skills to speak for themselves. This will impact the creation of self-determined and individualised supports for them.^28^ Three recommendations were made: (a) undertake more research on care determination situations, (b) enable greater involvement of self-advocacy groups in dialogues with providers, and (c) increase efforts on breaking down bias by research and ethics review boards on using persons with ID as informants.

## INDIVIDUALIZED SERVICES AND CLINICAL SUPPORTS

### Post-diagnostic support

Post-diagnostic supports (PDS) is the assistance provided from the time of diagnosis until when the person reaches end-of-life. Immediately after diagnosis is a time for communication between staff, carer, and person with ID, where the diagnosis and its implications are explained. It is a time for also anticipating and planning for future health changes (if these are expected to be significant) and the awareness for the need of making decisions about future support and care. PDS interventions also help with the management of idiosyncratic behavioural and psychological symptoms of dementia (PBSD)^29^ Three recommendations were made: (a) study the effectiveness of different non-pharmacological interventions, (b) develop guidelines for PDS applications by caregivers and support staff, (c) research the prevalence and nature of BPSD in adults with ID who develop dementia.

### Community dementia capable supports

Environmental changes increase disorientation and emotional instability. For persons with dementia, it is crucial to maintain meaningful relationships with family members and friends. For this reason, when dementia advances, community-centred housing is preferable over admission to nursing care facilities because the latter necessarily will change routines, involve restrictive care, and deprive social relationships. Dementia-related specialty housing reinforces positive supports, environmental adaptation, response to individualized needs, maintains autonomy, and a focus on quality care, person-centred approaches, and community integration. Three recommendations were made: (a) develop standards of care for community-based services that provide housing and other supports for persons with ID and dementia, (b) promote dementia-capable living environments in all places called ‘home’, and (c) prevent arbitrary changes in residence via fiat by government authorities.

### Advanced dementia

This term is reserved for when care needs or requirements and responses will differ from those present in earlier stage care, and reflect services more associated with greater debilitation and end-of-life. Identification of this dementia stage is far more complicated in people with ID due to the pre-existing intellectual impairment, concomitant health conditions, and confounding behaviours which may be similar to advanced dementia.^30^ Three recommendations were made: (a) support continued assessment for changes in disease progression, (b) encourage research directed at identifying more sensitive clinical tools for recognizing progression to late stage or advanced dementia, and (c) develop training in, and practice guidelines for, care practices with advanced dementia.

### End-of-life care

In this phase of dementia, physical care should be standardized with a focus on pain management, comfort, and relief from coincident conditions.^31^^,^ 32 In order to increase the use of palliative and hospice services, it is essential to reconcile ID providers´ and the family view. ‘Active support’ at end-of-life needs to be the foundation for guidelines for advanced care planning that respect later life rituals as well as consider consent and legal status-enabled planning. Three recommendations were made: (a) create a universal practice guideline on end-of-life supports, (b) encourage the use of such supports for end-of-life care in home settings, and (c) recognize variations in what ‘home’ may be like with respect to end-of-life care.

## ADVOCACY, PUBLIC IMPACT, AND FAMILY CAREGIVER ISSUES

### Terminology and the use of language

Although clinical or medical research follows structured descriptions of dementia or related terms to ID and dementia, other areas often lack precision.^33^ There is erratic use of language, despite established core methods and criteria in diagnosis, and insufficient comprehension of the dissimilarity within types of dementia. Consistency in terminology benefits the systematization of protocols, procedures and results, and aids in providing comprehensive communication. Three recommendations were made: (a) adopt a standardized list of terms for general use by providers and researchers, (b) standardize reporting to include key demographic and subject factors, and (c) promote positive imagery via non-stigmatizing language.

### Inclusion in national dementia plans and strategies

According to the WHO,^7^ all governments should develop and implement plans or strategies based on public policy and set goals for services, supports, and research related to dementia. These plans and strategies should consider peculiarities pertinent to the treatment of adults with ID and be converted into effective actions.^34^ Governments should involve adults with ID or their advocates during drafting of such official papers. Three recommendations were made: (a) include adults with ID in processes that create national plans, (b) advocate that governments provide supportive data related to ID for plan development, and (c) involve ‘self-advocates’ [persons with ID] in the development or review of policy documents and plans.

### Family caregivers

In Brazil, persons with ID usually live with their families as opposed to being institutionalized.^35^ In fact, the person providing care is more often a voluntary carer than a professional carer; the former is usually a family member such as a parent or sibling, but in some cases a neighbour or a friend. A common characteristic among them is that such carers are typically also older adults, with their own age-related limitations;^36^ indeed, many are middle-aged women.^13^ Most family carers may be overprotective and have fears that the person with ID might be neglected in an institution.^36^

The onset of dementia is an extra burden for carers, as persons with ID will lose daily living skills and exhibit adverse behavioural changes. Carers will experience uncertainty regarding access to information and supports available, as the more dementia progresses, the greater will be the difficulties in dealing with it.^37^ On this matter, three recommendations were delivered: (a) increase family support and enhance access to information and assistance with respect to family’s values, beliefs, ethnicity, and circumstances, (b) aid in ageing planning so that families are aware of steps to make appropriate decisions according to their own values and, if possible, valuing the person with ID’s opinion, and (c) reduce stress and provide resources to avoid and minimize the negative aspects of caregiving

## FINAL CONSIDERATIONS

Although there is a common component to ageing and dementia care across the globe, adults with ID still have idiosyncrasies in biopsychosocial aspects that require specialized attention. Consequently, it is crucial to determine what barriers may be present for continued community living after the diagnosis of dementia within the context of national public health and disability policies and availability of resources. Further, as noted by Scazufka et al.,^11^ in the Brazilian population, efforts directed at prevention of dementia should start early in life and continue through the life span – these same efforts need to encompass people with ID and be incorporated within national public health initiatives.

The Summit provided in-depth discussions on relevant topics pertinent to workers in ID, ageing, and dementia. The core goal of the Summit was that worldwide attention be given to this neglected matter. It was a fruitful meeting since there was a ‘meeting of the minds’ and numerous peer-reviewed articles were published and disseminated by open access regarding dementia and ID so as to give these notions broad coverage. The issues discussed should be considered the pillars that can influence and help frame national planning, public policy, clinical practice, services development, and research in this field.

We hope that this paper may lay the foundation for awareness about ID issues within Brazilian public health, ageing, and dementia communities. Beyond this, we urge researchers, clinicians and non-governmental organizations from the ID community to mobilize, support, and participate in the devising of a national plan for dementia. We envisage a stimulus for Brazilian non-governmental organizations to contribute strategies that promote citizenship, social inclusion, and rights of people with disabilities. However, for these programs to become effective in advancing disability rights there needs to be engagement of other sectors of society.^38^^,^ 39 We trust that when reviewing the output from the Glasgow Summit, both public and private non-governmental organizations will engage in dialogue to create legislation, initiatives, and public/private programs that can help adults with ID at risk or affected by dementia, as well as enabling their families and carers to have the resources to help them age with dignity.

When efforts are directed toward creating a national dementia plan (or regional or state plans) within Brazil, consideration should be given to including adults with ID, as noted by the Alzheimer’s Disease International^40^ and the WHO.^6^ More specifically, adults with ID should be included among the target groups of persons with a high risk for dementia and among adults with early-onset dementia. This is because such inclusion is an essential step to improve quality of life of persons with ID living with dementia and their families.

From the practical viewpoint, the Brazilian national plan should include initiatives such as:

- establishing regular dementia screening and follow-up after age 40 for adults at high risk (such as those with Down syndrome);
- adapting and translating into Portuguese, instruments for screening and diagnosis of dementia in persons with ID;
- initiating systematic and regular epidemiological studies investigating medical, social, and economic markers regarding people with ID living with dementia;
- developing and implementing training programs for the personnel who work with people affected by dementia and ID;
- expanding the options for community care of persons with dementia;
- engaging people with ID in proactive actions towards healthy ageing, such as adequate diet, physical exercise, stimulation of cognitive skills, and socialization; and
- investing in education of society aiming to raise awareness and understanding on how dementia affects adults with ID.

Brazil faces enormous challenges related to ageing and dementia; including consideration of dementia in adults with ID in whichever agenda is not just another responsibility. Dedicating attention to this cause will also improve the quality of life of two million Brazilians with ID, at risk of or living with dementia, and serve to allay apprehensions of family carers about the prospects of increased care demands in older age.

## Footnotes

* Usually people with ID are not encouraged to work but rather to obtain the Disability Pension (Benefício Assistencial; LOAS: Lei Orgânica da Assistência Social, Law 8.742/1993), a benefit for people with financial problems only, which is not granted for all people with intellectual disability in Brazil. In some cases, the pension supports the entire family.
